# The burden of back pain on hospital staff in a maximum care facility in Germany – a survey

**DOI:** 10.1016/j.bas.2025.104338

**Published:** 2025-07-23

**Authors:** Josina Straub, Melanie Ardelt, Kristina Gerhardinger, Lisa Klute, Jonas Krueckel, Markus Rupp, Volker Alt, Siegmund Lang

**Affiliations:** a Clinic und Policlinic for Trauma Surgery, University Hospital Regensburg, Germany; b Department for Orthopaedics and Traumatology, University Hospital Krems, Austria; c Center for Regenerative Medicine and Orthopaedics, Danube University Krems, Austria; d Department of Trauma, Hand and Reconstructive Surgery, University Hospital Giessen, Germany

**Keywords:** Back pain, Working conditions, Healthcare workers, Risk factors

## Abstract

**Purpose:**

Back pain is a prevalent musculoskeletal condition affecting individuals across various professions. It is a significant cause of disability and reduced quality of life. The aim of this study was to determinate the prevalence of back pain and identify its associated risk factors among clinical staff members.

**Methods:**

This study examines back pain among hospital-staff at a maximum-care hospital in Germany through a questionnaire distributed to all employees at a University Hospital between September and December 2023.

**Results:**

A total of 739 employee questionnaires were returned and evaluated, comprising 508 fully completed and 231 partially completed questionnaires. Seventy-seven percent of participants were female, and the average age was 40.5 years. Back pain-related reduced work performance was reported by 57.5 %. Lifetime lower back pain was indicated by 72.1 %. Mean pain ratings on the VAS were 7.5 ± 2.2 for lifetime pain and 6.2 ± 2.2 for the last 12 months. Female gender (OR = 1.5; p = 0.003), living in a partnership (OR = 1.4; p = 0.02), chronic illnesses (OR = 6.4; p = 0.01) and excessive workload (OR = 3.0; p = 0.05) were identified as risk factors, whereas membership in a sports club (OR = 0.55; p = 0.05) was identified as preventive factor. Participants that reported an aggravation of their back pain the COVID-19 pandemic were 6.4 times likely to report back pain during the last 12 months (p = 0.01).

**Conclusion:**

Back pain is a widespread condition among healthcare workers, leading to reduced performance and significant healthcare utilization. Employers should prioritize preventive measures and workplace ergonomics to mitigate this burden and enhance employee well-being and productivity.

**Level of evidence:**

III, survey.

## Highlights

• High burden of back pain among health-care workers leading to reduced performance.
• Risk factors: COVID-19 pandemic, female gender, partnership and work overload.
• Preventive factors: ergonomic workplace and membership in a sports club.

## 1 Introduction

Back pain is a common musculoskeletal disorder affecting individuals in various professions, contributing to disability and reduced quality of life (A et al., 2020). The Global Burden of Disease study found that 577 million people had lower back pain in 2017 accounting for 64.9 million years lived with disability, making lower back pain a leading global cause of disability (A et al., 2020; Hartvigsen et al., 2018). Notably, in middle-income countries the number of disability-adjusted life-years for low back pain increased by 54 % since the year 1990 (Hartvigsen et al., 2018). In the United States, the one-year prevalence of back pain ranges from 10.3 % to 56 %, with a lifetime prevalence up to 80 % (Hoy et al., 2012). In addition, it is the most common reason for medically certified leave and early retirement in Europe, surpassing all other musculoskeletal condition (Buchbinder et al., 2020).

Among healthcare professionals, clinical staff members, are particularly susceptible to developing back pain due to nature of their work (Rezaei et al., 2012). These individuals often perform physically demanding tasks, such as patient handling, repetitive movements and prolonged standing or sitting, all of which can contribute to the development of back pain (Rezaei et al., 2012). Psychosocial factors, such as job dissatisfaction, stress and lack of social support, may also contribute to the onset and progression of back pain as it is linked to multifaceted interaction (Choi et al., 2021; Cowell et al., 2018). These aspects may contribute to a cycle of pain and incapacity (Cowell et al., 2018). To ensure effective management, it is crucial to accurately identify the underlying causes of pain and consider potential non-nociceptive factors contributing to establish a precise diagnosis and determine the most appropriate treatment approaches (Conger et al., 2022).

Therefore, this study aimed to investigate the prevalence of back pain and identify its associated risk factors among clinical staff members, including administrative and nursing staff as well as doctors. By surveying this specific group, we aimed to gain insights into the occupational and individual factors contributing to back pain in the healthcare setting. Understanding these factors is crucial for implementing targeted interventions to promote better back health among clinical staff and improve patient care.

## 2 Materials and methods

### 2.1 Data collection

This research analyses data of back pain among the hospital staff at a maximum care facility in Germany, Bavaria. For this purpose, a questionnaire was distributed to all employees at the university hospital in Regensburg, with participation open from September to December 2023. In addition to nurses and physicians, employees from administration as well as other healthcare-related professions, such as physiotherapy, occupational therapy and speech therapy, were surveyed. Anthropometric data, comorbidities, working conditions, working time, type of profession, pain intensity based on the Numerical Rating Scale (NRS), pain localization and the prevalence of pain in the past 12 months according to localization were recorded. The NRS was used to achieve an objective and comparable assessment (Karcioglu et al., 2018). Additionally, the number of sick days, performed diagnostic procedures, potential methods for pain alleviation and workplace adjustments implemented by the employer were documented.

The questionnaire was distributed to over 2000 employees of the University Hospital Regensburg. The relevant questions from the employee questionnaire are presented in the following table (Table 1).

**Table 1:** Question from the employee questionnaire.

1. Have you ever experienced pain in the following regions: cervical, thoracic, lumbar or multiple regions of the spine?
2. Have you experienced pain in the following regions within the last 12 months, 3 months or currently: cervical, thoracic, lumbar or multiple regions of the spine?
3. How frequently did you experienced back pain over the past year: less than 6 weeks, 6–12 weeks or more than 12 weeks?
4. How severe was your most intense back pain: lifetime, currently, last 12, 6 or 3 months?
5. How severe was your average back pain: lifetime, currently, in the last 12, 6 or 3 months?
6. How often in life have you experienced daily back pain lasting over 3 months?
7. Do your back pain radiate?
8. Have back pain ever caused you to limit daily activities?
9. How many years of professional do you have?
10. On average, how many hours do you work per week?
11. Have you ever stopped working due to back pain?
12. Do you feel that back pain limits your professional performance?
13. What percentage of work involves direct patient care?
14. Do you have to lift heavy objects (>7 kg) during work?
15. Do you have to work in unergonomic position ?
16. How often do you feel stressed during work?
17. How often do you feel overwhelmed during work?
18. Which of the following ergonomic adjustments do you use at workplace: height-adjustable desk, ergonomic office chair, height-adjustable monitor, personalized protective equipment?
19. Has ergonomic workplace adjustment improved back pain?
20. Which workplace-related measures do you consider effective in reducing back pain?
21. Were you sick leave due to back pain last year?
22. Have you ever sought medical treatment for back pain?
23. Have you ever received examinations for back pain: X-ray, CT, MRI?
24. Have you been diagnosed with a structural cause for back pain?
25. Have you ever undergone physiotherapy for back pain?
26. Do you take pain medication for back pain?
27. Have you ever been hospitalized for back pain?
28. Which specialty do you trust for back pain treatment: family doctor, orthopaedics, neurosurgeon, alternative medicine?
29. Was your spine injured during an accident?
30. How many hours per week do you exercise on average?
31. Are you a member of a sport club?
32. Do you suffer from chronic conditions?
33. Have you noticed a worsening of back pain during the Covid-19 pandemic?
34. How much do you pay attention to maintaining a healthy diet?
35. How often do you consume alcohol?
36. Do you take dietary supplements regularly?
37. How old are you?
38. Are you in a relationship?
39. Are you living with a child?
40. Your professional group: nurse, physicians, administration
41. Please specify your working hours: full-time or part-time?
42. What is your level of education?

MRI = magnetic resonance imaging, CT = computertomography.

### 2.2 Statistical analysis

The data were investigated to determine the prevalence of back pain and identify its associated risk factors among all clinical staff members, including administrative and nursing staff as well as doctors. By surveying this specific group, we aimed to gain insights into the occupational and individual factors contributing to back pain in the healthcare setting. The statistical analysis was done using the statistical package SPSS (IBM SPSS Statistics, version 30.0.0, Zürich, Swiss). Categorical variables were presented in terms of frequency and percentage. Descriptive Statistics were calculated for the characteristics of back pain among the healthcare workers. Continuous variables are presented in mean and standard deviation, categorical variables in number of observations and frequency. The odds ratio (OR) was utilized to assess risk and preventive factors associated with back pain over the past 12 months. Furthermore, the analysis included the evaluation of risk and preventive factors for the different regions of the spine. The significance level was defined at 5 %.

To identify significant predictors of self-reported back pain within the past 12 months, we performed a multivariable logistic regression analysis using a forward stepwise selection procedure. Variables were entered into the model based on statistical significance (entry criterion: p < 0.05), starting with the predictor most strongly associated with the dependent variable. The dependent variable was binary (presence vs. absence of back pain within 12 months). Variables with low prevalence or causing quasi-complete separation were excluded from the final model to avoid unstable coefficient estimation. To assess the goodness-of-fit of the final model, we applied the Hosmer-Lemeshow test. To reduce confounding and selection bias, we included a broad set of covariates informed by prior literature and theoretical considerations, including sociodemographic, behavioral and health-related covariates known or plausibly linked to both the exposure and the outcome.

## 3 Ethical approval

The study was conducted in accordance with the ethical standards of the Declaration of Helsinki. A dedicated ethical approval was not required according to the local ethics committee, as the study did not involve identifiable data or interventions necessitating ethical oversight by the ethics committee. Informed consent was obtained from all participants. Further, approval for the survey was obtained from the Medical Director and the Nursing Directorate and it was conducted in collaboration with the Quality Management Department.

## 4 Results

A total of 739 employee questionnaires were returned and evaluated, comprising 508 fully and 231 partially completed questionnaires. The average age was 40.5 years and 77 % were female. Healthy diet was important for 60.2 %. Chronic conditions were reported by 27.1 % and 22.3 % indicated worsening of their back pain during the COVID-19 pandemic. An additional analysis was performed to investigate factors contributing to the exacerbation of back pain during the COVID-19 pandemic: Increased psychological distress was identified as a contributing factor by 24.8 %, increased workload by 24.4 % and reduced physical activity by 25.4 %. The detailed anthropometric data are presented in Table 2.

**Table 2:** Anthropometric data on patient collective.

Characteristics	N or average	% or SD
age	40.5	11.4
female	386	77.0
living in a partnership	388	77.3
household with children	171	34.1
dietary supplements	141	28.1
*healthy diet*		
never	4	0.1
rare	19	3.9
occasionally	134	26.8
often	305	60.2
always	40	8.0
*alcohol consumption*		
never	70	14
rare	247	49.2
occasionally	165	33.0
often	18	3.7
daily	2	0.1
full-time employment	341	68.2
chronic mental illness	48	9.7
chronic disease	136	27.1
*concomitant symptoms*		
tension headache	263	23.1
sleeping disorders	220	19.3
visual impairment	61	5.4
aggression	90	7.9
tension	189	16.5
anxiety	44	3.9
lack of concentration	112	9.8
listlessness	142	12.5
sports club membership	207	41.2
weekly sports lessons	3.55	3.91
aggravation during Covid-19 pandemic	112	22.3
*reasons for aggravation during Covid-19*		
no ergonomic workplace	12	4.0
psychological distress	75	24.8
increased workload	74	24.4
financial concerns	19	6.3
reduced social interactions	43	14.2
decreased physical activity	77	25.4
others	3	0.9
*educational degree*		
none	3	0.6
secondary school	230	40.7
a-level	99	19.6
university degree	106	21
promotion/habilitation	51	10.1
*professional group*		
administration	197	43.8
nursing	72	16.0
OR-nursing	32	7.2
operative physicians	24	5.3
no operative physicians	23	5.1
others	102	22.6

N = number.

The average work experience was 15.5 years, with an average weekly working time of approximately 35 h. Reduced work performance due to back pain was reported by 57.5 % and 46.7 % complained of an unergonomic posture at the workplace. Frequent stress at work was experienced by 74.9 % and excessive demands at workplace was reported by 25.0 %. A total of 29.7 % failed to achieve an ergonomic adjustment of their workplace. As a result of ergonomic workplace adjustments, 5.9 % experienced complete pain relief or a significant reduction in pain intensity (Table 3).

**Table 3:** Workplace-specific characteristics.

Characteristics	N or Median	% or SD
professional experience (years)	15.5	10.9
weekly working hours	35	11.0
termination of service due to back pain	174	35.2
reduced performance due to back pain	285	57.5
sedentary work (hours)	6.8	3.2
direct patient contact (hours)	4.5	3.8
heavy lifting activity	194	39.5
unergonomic posture	233	46.7
*stress at workplace*		
never	1	0.2
rare	121	24.9
frequently	206	42.3
very frequently	150	30.8
always	9	1.8
*excessive demands at workplace*		
never	56	11.3
rarely	311	62.6
frequently	102	20.5
very frequently	27	5.4
always	1	0.2
*ergonomic adjustment*		
none	208	29.7
height-adjustable desk	35	5.0
ergonomic keyboard	60	8.6
height-adjustable computer	193	27.6
ergonomic chair	134	19.1
individualized protective clothing	31	4.4
others	39	5.6
*improvement after ergonomic adjustment*		
complete pain relief	3	0.5
significant	27	5.4
something	61	12.2
none	162	32.3
no adjustment received	248	49.5

N = number, SD = standard deviation.

**Fig. 1:**
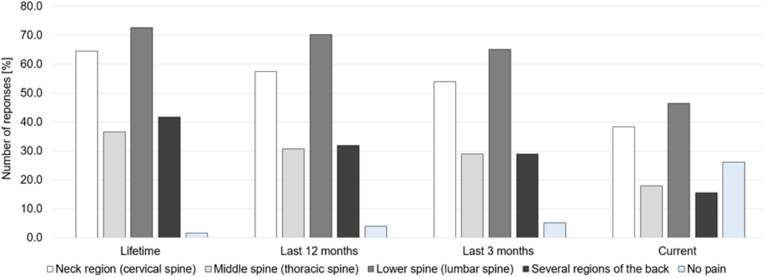
Self-reported prevalence of back pain during lifetime, in the last 12 months, in the last 3 months and currently.

Lifetime lower back pain was indicated by 72.1 %. Pain in the lumbar spine was most reported followed by pain in the cervical spine. Additionally, the prevalence lifetime, after 3 and 12 months and currently stayed equal. Within the last 12 months, 58.1 % experienced cervical back pain, 30.8 % reported thoracic back pain and 70.0 % suffered from lumbar back pain. Furthermore, 32.1 % reported pain in multiple spinal regions, while only 4.2 % reported an absence of back pain in the last 12 months (Fig. 1).

For their back pain, 77.9 % sought medical treatment including physiotherapy, painkillers or alternative methods such as acupuncture. Due to back pain, 20.6 % were on medically certified leave for up to one week, while 3 % were on medically certified leave for more than 30 days. Approximately 70 % of respondents underwent diagnostic imaging for their back pain, with around 25 % undergoing X-ray and magnetic resonance imaging (MRI), respectively. A consultation of orthopedic specialist for back pain was considered to be beneficial by 47.0 %. Physical therapy has been received by 76.4 %. Additionally, 5.6 % had been hospitalized due to back pain. Structural causes for back pain were detected in 35.1 % through imaging (Table 4).

**Table 4:** Treatment strategy for back pain.

Characteristics	N	%
*medically certified leave*		
1–3 days	54	10.8
4–7 days	49	9.8
8–30 days	41	8.2
>30 days	15	3.0
none	343	68.2
medical treatment	388	77.9
*examination*		
X-ray	165	25.2
CT	75	11.5
MRI	173	26.4
none	234	35.7
others	8	1.2
structural cause back pain	176	35.1
pain radiation	329	65.7
physiotherapy	376	76.4
*consumption of painkillers*		
never	124	24.7
rarely	161	32.1
occasionally	168	33.5
regularly	43	8.6
daily	6	1.1
hospitalization	28	5.6
*specialist for treatment*		
family doctor	184	24.4
emergency room	3	0.4
orthopaedics	355	47.0
neurosurgery	79	10.5
alternative medicine	83	11.0
others	51	6.8
*positive experience for back pain relief*		
yoga	197	35.1
meditation	57	10.1
imagination	5	0.8
progressive muscle relaxation	113	20.1
autogenic training	43	7.6
biofeedback	3	0.5
acupuncture	56	9.9
others	89	15.9

N = number, SD = standard deviation. CT = computertomography, MRI = Magnetic Resonance Imaging.

**Fig. 2:**
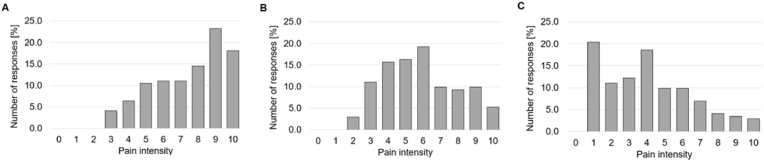
Pain intensity ratings from 0 = no pain to 10 = most intense pain for (A) the most severe pain, (B) the average pain, and (C) the current pain.

Mean pain VAS ratings yielded 7.5 ± 2.2 for the strongest pain experienced during lifetime, 6.2 ± 2.2 for the pain during the last 12 months, and 5.5 ± 2.4 during the last 3 months. The current pain was rated as 3.9 ± 2.6 on average (Fig. 2).

A binary logistic regression analysis was conducted using forward stepwise selection to identify significant predictors of the outcome variable. In total, five predictors were entered into the model across four steps: physiotherapy, sports, tension headache, living in a partnership. In the final model, significant predictors included sports (B = 1.68, p = 0.001), physiotherapy (B = −2.32, p < 0.001), living in a partnership (B = −1.43, p = 0.006) and tension headache (B = 1.61, p = 0.007) (Table 5). Aggravation during COVID-19 initially entered the stepwise model but was excluded from the final model due to model instability and non-significance (B = −18.2; p = 0.99), indicating quasi-separation.

**Table 5:** Logistic regression results using forward stepwise selection across four steps.

Characteristics	Regression coefficients (B)	p-value	OR
sports	1,68	0.001	5.41
physiotherapy	−2.32	<0.001	0.38
living in a partnership	−1.43	0.006	0.24
tension headache	−1.6	0.007	0.20

Model fit was evaluated using the Hosmer–Lemeshow goodness-of-fit test, which assesses agreement between observed and expected event rates across risk deciles. The non-significant result (χ^2^ = 5.17, p = 0.52) indicates acceptable model calibration and supports the adequacy of the logistic regression model.

Risk factors for the prevalence of back pain within the past 12 months were analyzed for the entire spine as well as for the various regions. For the entire spine, female gender (OR = 1.5; p = 0.003), living in a partnership (OR = 1.4; p = 0.02) and chronic illnesses (OR = 6.4; p = 0.01) were identified as risk factors. Additionally, the perception of symptom aggravation during the COVID-19 pandemic (OR = 6.8; p = 0.01) and excessive workload (OR = 3.0; p = 0.05) were identified as risk factors, whereas membership in a sports club (OR = 0.55; p = 0.05) was identified as potential preventive factor for back pain. Alcohol consumption (OR = 0.9; p = 0.75), psychological diseases (OR = 2.5; p = 0.11) and mental health disorders (OR = 2.5; p = 0.11) demonstrated no statistical significance for their tendency as risk factors (Table 6).

**Table 6:** Risk and preventive factors for back pain within the last 12 months.

Characteristics	OR	p-value	95 %-CI
**female**	**1.5**	**0.003**	**1.0–2.2**
**partnership**	**1.4**	**0.02**	**0.9–2.0**
household with child	0.8	0.32	0.5–1.2

**chronic disease**	**6.4**	**0.01**	**0.9–43.8**
psych disease	2.5	0.11	0.7–2.8
**aggravation during Covid-19**	**6.8**	**0.01**	**3.4–7.8**

alcohol consume	0.9	0.75	0.5–1.5
dietary supplements	2.1	0.11	0.7–6.3
healthy diet	1.3	0.09	0.8–1.9

heavy lifting	1.1	0.72	0.63–1.97
ergonomic workplace	1.4	0.24	0.77–2.4
stress at work	1.0	0.65	0.8–1.4
**excessive work demand**	**3.0**	**0.05**	**0.8–11.5**

yoga	1.8	0.09	0.8–1.2
mediation	1.3	0.7	0.3–5.0
progressive muscle relaxation	1.8	0.28	0.6–5.1
**membership sports club**	**0.55**	**0.05**	**0.4–0.7**

OR = odds ratio, CI = confidence interval.

A subgroup analysis was performed to investigate risk and preventive factors for various spinal regions over a 12-month period. Female gender (OR = 1.7; p < 0,001) and heavy lifting (OR = 1.4; p = 0.01) were identified as risk factors for cervical back pain within the past 12 months. In contrast, the use of dietary supplements (OR = 0.6; p = 0.01) was identified as a preventive factor for cervical back pain. Membership in a sports club (OR = 0.8; p = 0.45) and living in a partnership (OR = 0.9; p = 0.61) were potential preventive factors but did not reach statistical significance (Table 7).

**Table 7:** Risk and preventive factors for cervical back pain within 12 months.

Characteristics	OR	p-value	95 % - CI
**female**	**1.7**	**<0.001**	**1.3–2.4**
partnership	0.9	0.61	0.8–1.1
household with child	1.1	0.71	0.8–1.4

chronic disease	0.7	0.02	0.5–0.9
psych disease	0.9	0.62	0.5–1.5
aggravation during Covid-19	1.0	0.90	0.7–1.9

alcohol consume	1.0	0.73	0.7–1.2
**dietary supplements**	**0.6**	**0.01**	**0.5–0.9**
healthy diet	0.9	0.07	0.8–1.1

**heavy lifting**	**1.4**	**0.01**	**1.1–1.7**
ergonomic workplace	1.1	0.35	0.9–1.3
stress at work	1.1	0.51	0.9–1.2
excessive demand	0.9	0.78	0.7–1.4

sports	0.9	0.48	0.7–1.2
yoga	0.8	0.13	0.6–1.1
mediation	0.8	0.29	0.4–1.3
progressive muscle relaxation	0.8	0.33	0.6–1.2
membership sports club	0.8	0.45	0.7–1.2

OR = odds ratio, CI = confidence interval.

For thoracic back pain within the past 12 months no identifiable risk factors were found, whereas ergonomic workplace adjustments (OR = 0.8; p = 0.04) and progressive muscle relaxation (OR = 0.7; p = 0.04) were identified as preventive factors (Table 8).

**Table 8:** Risk and preventive factors for thoracic back pain within 12 months.

Characteristics	OR	p-value	95 % - CI
female	1.2	0.35	0.8–1.7
partnership	0.9	0.27	0.8–1.1
household with child	1.2	0.16	0.9–1.6

chronic disease	0.8	0.10	0.6–1.1
psychological disease	0.6	0.07	0.4–1.1
aggravation during Covid-19	1.0	0.45	0.9–1.2

alcohol consume	1.1	0.58	0.8–1.4
dietary supplements	0.9	0.62	0.7–1.3
healthy diet	1.1	0.72	0.8–1.2

heavy lifting	0.8	0.12	0.6–1.1
**ergonomic workplace**	**0.8**	**0.04**	**0.67–1.0**
stress at work	1.1	0.37	0.9–1.2
excessive demand	1.1	0.09	0.6–1.2

sports	1.0	0.62	0.8–1.4
yoga	0.8	0.09	0.7–1.0
mediation	0.8	0.33	0.5–1.3
**progressive muscle relaxation**	**0.7**	**0.04**	**0.5–1.0**
membership sports club	0.9	0.78	0.7–1.2

membership sports club	0.9	0.78	0.7–1.2

OR = odds ratio, CI = confidence interval.

For lumbar back pain within the past 12 months no significant risk or protective factors could be determined. Female gender (OR = 1.1; p = 0.85), membership in a sports club (OR = 1.0; p = 0.65), aggravation due to the COVID-19 pandemic (OR = 0.9; p = 0.92), ergonomic workplace adjustments (OR = 1.1; p = 0.76) and stress at work (OR = 1.0; p = 0.83) could not be classified either as risk or preventive factors (Table 9).

**Table 9:** Risk and preventive factors for lumbar back pain within 12 months.

Characteristics	OR	p-value	95 % - CI
female	1.1	0.85	0.9–1.2
partnership	0.9	0.63	0.8–1.1
household with child	1.1	0.43	0.8–1.4

chronic disease	0.9	0.32	0.6–1.2
psychological disease	0.8	0.43	0.4–1.4
aggravation during Covid-19	0.9	0.92	0.7–1.2

alcohol consume	0.9	0.28	0.7–1.2
dietary supplements	1.0	0.82	0.7–1.3
healthy diet	0.9	0.10	0.8–1.0

heavy lifting	0.8	0.89	0.8–1.2
ergonomic workplace	1.1	0.76	0.8–1.3
stress at work	1.0	0.83	0.9–1.1
excessive demand	1.0	0.95	0.7–1.4

sports	1.1	0.14	0.9–1.5
yoga	1.1	0.36	0.9–1.4
mediation	0.7	0.34	0.4–1.3
progressive muscle relaxation	1.1	0.47	0.8–1.6
membership sports club	1.0	0.65	0.8–1.3

OR = odds ratio, CI = confidence interval.

Additionally, an analysis was performed to assess the prevalence of back pain, the prevalence associated risk factors and mean pain prevalence across the various professions within the last 12 months. The mean VAS pain scores over the past 12 months were 3.4 ± 1.9 for physicians, 5.4 ± 1.9 for nursing staff, and 5.2 ± 2.1 for administrative personnel. Regular heavy lifting at work was reported by 78.8 % of nursing staff and 36.7 % of physicians. Additionally, approximately one-quarter of all professional groups indicated experiencing excessive workload. Detailed data on pain prevalence by region, mean VAS pain scores and risk factors for each professional group are presented in Table 10.

**Table 10:** Prevalence of back pain, mean pain intensity and the prevalence of risk factors in various professional group over the last 12 months.

Prevalence pain last 12 months (n)					
Profession	Cervical	Thoracic	Lumbar	Total spine	Pain intensity
physician	27 (55.1 %)	12 (24.5 %)	25 (51 %)	12 (24.5)	3.4 (±1.9)
nurse	88 (55 %)	49 (30.6 %)	111 (69.4 %)	49 (30.6 %)	5.4 (±1.9)
administration	206 (68.9 %)	83 (27.8 %)	197 (65.9 %)	95 (31.8 %)	5.2 (±2.1)

Risk factors (n)						
Profession	Female	Partnership	Excessive demand	Aggravation during Covid-19	Stress at work	Heavy lifting
physician	26 (53.1 %)	40 (81.6 %)	12 (24.5 %)	3 (6.1 %)	40 (81.6 %)	18 (36.7 %)
nurse	123 (76.9 %)	114 (71.3 %)	47 (29.4 %)	42 (26.3 %)	136 (85 %)	126 (78.8 %)
administration	243 (81.3 %)	234 (78.3 %)	71 (23.7 %)	67 (22.4 %)	199 (66.6 %)	50 (16.7 %)

n = number.

## 5 Discussion

In this survey among 736 health-care workers we identified a high burden of back pain, accompanied by a plethora of concomitant symptoms, leading to a relevant reduction in performance. Several risk factors for the prevalence of back pain during the last 12 months were identified, of which the most pronounced were the perception of symptoms aggravation during COVID-19 pandemic, female gender, living in a partnership and excessive demand at work. The few preventive factors included an ergonomic workplace, membership in a sports club and activities like progressive muscle relaxation.

### 5.1 Anthropometric data and work-place characteristics

Back pain is a prevalent musculoskeletal condition affecting individuals across various professions causing disability and reduced quality of life (A et al., 2020). Healthcare professionals are especially prone to back pain due to the physical demands of their roles (Cowell et al., 2018). Their responsibilities often involve activities like patient handling, repetitive motions and extended periods of standing or sitting known as increased risk for developing back pain (Cowell et al., 2018). Chronic low back pain is linked to a multifaceted interaction of physical, behavioral, psychological as well as lifestyle factors (Cowell et al., 2018). These aspects may contribute to a cycle of pain and incapacity (Cowell et al., 2018). The mean age in our cohort was 40.5 years, which is younger compared to the reported 46 years in the literature (Bayartai et al., 2023; Virkkunen et al., 2022). Consistent with other studies, our cohort demonstrated a higher rate of women complaining about back pain with 77 % (Hoy et al., 2012; Rufa et al., 2022; Shrestha et al., 2021).

Strikingly, 57.5 % described a reduced work performance due to back pain, which is comparable with literature (Russo et al., 2021). Lumbar back pain was the most prevalent region for back pain, affecting 72.3 % of participants. This aligns with rates reported in the literature (Mroczek et al., 2020). Comparable to our cohort, Pinto et al. reported a current prevalence of lumbar back pain of 65.2 % among nurses (Corrêa Pinto et al., 2021). Nevertheless, the prevalence of lower back pain in the last 3 months was significantly lower at 22.4 %, compared to 65.1 % in our cohort (Corrêa Pinto et al., 2021). Heymans et al. reported 33.8 % of participants in their study on non-specific lower back pain among workers experienced pain radiating into the legs (Heymans et al., 2010). However, our survey revealed nearly twice this prevalence, with 65.7 % reporting radiating pain.

In our study, approximately a quarter of all professional groups reported work-related demand. Additionally, 85 % of nurses and 81.6 % of physicians experienced stress at work and 78.8 % of nurses complained about regularly heavy lifting. For nursing staff, the prevalence of heavy lifting was consistent with values reported in the literature (Holtermann et al., 2013).

Data on the relationship between back pain and the adherence to a healthy diet or the use of dietary supplements is limited in literature (Virkkunen et al., 2022; Freimann et al., 2013). Virkkunen et al. reported a smoking prevalence of 28.3 % within their cohort, while further information remains sparse in literature (Virkkunen et al., 2022). In our cohort, 36.8 % reported regular alcohol consumption, 61 % prioritized maintaining a healthy diet and 28.1 % indicated regular use of dietary supplements. Various studies have reported an association between smoking, alcohol consumption and the occurrence of back pain (Ferreira et al., 2013; Kardouni et al., 2016). Smoking behaviour and its potential association with back pain was not assessed in the current study.

In addition to back pain, 27.1 % reported having a chronic illness. Mental health conditions were detected by 9.7 %, with a prevalence of 8.0 % among women. According to Virkkunen et al. the prevalence of depressive symptoms among female employees in the healthcare demonstrated 28 % (Virkkunen et al., 2022). Additionally, 23.1 % of participants reported suffering from tension headache and 19.3 % complained of sleep disorder. The prevalence of sleep disturbances is described in the literature slightly lower with approximately 15 (Ramond et al., 2011).

### 5.2 Risk factors

While the majority of patients with back pain recover on their own, around 10 % will eventually acquire chronic back pain (Heymans et al., 2010). The most commonly noted risk factors for persistent back pain were psychosocial conditions and work-related concerns, such as lifting heavy and challenging working situations (Nieminen et al., 2021). To the best of our knowledge, our study is one of the few to investigate risk and preventive factors for back pain among healthcare workers, addressing both the entire spine as well as its specific regions. In our analysis, female gender (OR = 1.5; p = 0.003), living in a partnership (OR = 1.4; p = 0.02), chronic illnesses (OR = 6.4; p = 0.01), the perception of symptom aggravation during the COVID-19 pandemic (OR = 6.8; p = 0.01) and excessive work demand (OR = 3.0; p = 0.05) were risk factors, whereas membership in a sports club (OR = 0.55; p = 0.05) was identified as preventive factor for back pain of the entire spine.

Several studies have investigated risk and preventive factors for back pain (Freimann et al., 2013; Matsudaira et al., 2011; Zouch et al., 2022). Freimann et al. mentioned poor self-rated health, emotional exhaustion and stressful physical activities at work as risk factors among nurses (Freimann et al., 2013). General and occupational stress were reaffirmed as significant risk factors for both back pain and sleep disorders. In contrast, physical workload, particularly suboptimal posture, exhibited a stronger association with back pain than with sleep disorders (Hämmig, 2020). However, in our study stress at work (OR = 1.0; p = 0.65) could not be identified as risk factors, whereas excessive demand at work (OR = 3.0; p = 0.05) as well as chronic illnesses (HR = 6.4; p = 0.01) were risk factors for the entire spine. For cervical back pain, neither stress at work (OR = 1.1; p = 0.51) nor excessive demand (OR = 0.9; p = 0.78) could be identified as risk factors. While work-related stress was identified as risk factor in several studies (Rezaei et al., 2012), the absence of stress as a risk factor in our cohort prompts the question of whether the consistently high stress levels observed are a result of regular exposure to severely injured patients in a maximum care hospital or due to the inclusion of administrative staff in the analysis. Furthermore, elevated levels of psychological fatigue may adversely affect both the physical and mental health of individuals in the workplace, potentially increasing the risk of injury among medical staff (Gander et al., 2019).

Interestingly, the association between the prevalence of back pain and the response to the question of whether symptoms worsened during the COVID-19 pandemic (OR = 6.8; p = 0.01) suggests that individuals who attribute their pain to external factors may be more prone to experiencing back pain (Shrestha et al., 2021). The hypothesis posits individuals attributing their pain to external stressors may be at greater risk of developing chronic symptoms (Shrestha et al., 2021). This aligns with psychological theories of stress management. Individuals with an external locus of control, characterized by attributing challenges to external factors, are more likely to experience prolonged distress and delayed recovery (Behisi et al., 2013). In contrast, those with an internal locus of control, who believe in their capacity to influence outcomes, demonstrate greater resilience to stress and improved health outcomes (Behisi et al., 2013).

Behisi et al. performed an analysis about back pain among health-care workers in Saudi Arabia. Hereby, female gender (OR = 1.9, CI = 1.3–2.7) was as risk factor for back pain, concordant with our findings (Behisi et al., 2013). Furthermore, female gender (OR = 1.7; p < 0.001) was identified as a risk factor for cervical spine pain.

Several studies identified heavy lifting as a risk factor (Bin Homaid et al., 2016; Khumalo and Haffejee, 2022). In our analysis, heavy lifting was as well identified as risk factor for cervical back pain (OR = 1.4; p = 0.01). This highlights the importance of enrolling health-care workers in educational intervention programs focused on proper lifting techniques and strategies for managing various situations (Bin Homaid et al., 2016).

Furthermore, living in a partnership (OR = 1.4; p = 0.02) was identified as a risk factor for the entire spine in our cohort study. Almaghrabi et al. indicated a higher prevalence of lower back pain among married participants compared to single, divorced and widowed nurses (Almaghrabi and Alsharif, 2021). Cultural factors may contribute to this, as particulary married women are often exposed to physically demanding household tasks, in addition to their work responsibilities. However, no significant relation between prevalence of lower back pain and marital status was found in literature (Almaghrabi and Alsharif, 2021; Sun et al., 2007).

### 5.3 Preventive factors

Our analysis identified membership in a sports club (OR = 0.55; p = 0.05) as preventive factor for back pain of the entire spine as well as ergonomic workplace adjustments (OR = 0.8; p = 0.04) and progressive muscle relaxation (OR = 0.7; p = 0.04) as preventive factors for thoracic back pain. In a Saudi Arabian study on back pain among healthcare workers, regular physical activity was highlighted as a significant preventive factor for back pain. Membership in a sports club is likely associated with increased physical activity, potentially leading to improved core stability and a subsequent reduction in back pain (Alnaami et al., 2019; Knezevic et al., 2021). These findings align with the literature, which also recognizes ergonomic adjustments as a protective factor (Rezaei et al., 2012). Implementing ergonomic workplace adjustments in additional areas should be prioritized to further reduce the prevalence of back pain, contributing to a decrease in medically certified leave and associated healthcare costs.

While several variables (e.g., female sex, chronic disease, COVID-19-related aggravation) showed significant associations in univariate analyses, they did not enter the final multivariable model due to collinearity or lack of additional predictive value. The stepwise procedure thus highlights the most robust independent predictors, though univariate findings remain clinically informative.

## 6 Limitations

This study relies on self-reported data, subject to recall bias, and was conducted in a single hospital, limiting generalizability. This design precludes longitudinal follow-up, limiting inferences regarding pain chronicity. The response rate below 50 % introduces potential non-response bias, and the cross-sectional design precludes causal inferences. Additionally, while logistic regression was used to adjust for key confounders such as age, sex and chronic conditions, residual confounding from unmeasured factors cannot be ruled out. Due to the exploratory approach and the number of variables analyzed, the potential for spurious associations cannot be fully excluded. Results should be interpreted in light of this and regarded as indicative rather than conclusive. Key factors like BMI and smoking were not assessed, as their inclusion as well as a more detailed assessment of pandemic-related workload changes was declined by the Nursing Directorate due to expressed concerns. In addition, the COVID-19–related aggravation of symptoms referred specifically to participants' subjective perception of symptom changes during the pandemic rather than clinically verified changes in symptom severity. Future multicenter studies with objective measures are needed.

## 7 Conclusion

Back pain is a widespread condition among our cohort of healthcare workers, leading to reduced performance and significant healthcare utilization. Employers should prioritize preventive measures and workplace ergonomics to mitigate this burden and enhance employee well-being and productivity.

## Informed consent

Not necessary.

## Data availability statement

The data that support the findings of this study are not openly available due to reasons of sensitivity and are available from the corresponding author upon reasonable request. Data are available upon reasonable request by contacting the corresponding author via email, with data sharing facilitated through secure encryption protocols.

## Authors' contribution

The manuscript was created by JS and SL. JS, MA and SL performed the statistical analysis and designed the study. JS, SL, MR, KG, LK, VA, JK and MA conceived of the study, helped to draft the manuscript and participated in its design. All authors read and approved the final manuscript.

## Funding

This research did not receive any specific grant from funding agencies in the public, commercial, or not-for-profit sectors.

## Declaration of competing interest

The authors declare that they have no known competing financial interests or personal relationships that could have appeared to influence the work reported in this paper.
